# Editorial: Cheminformatics Approaches in Drug Discovery for Neglected Tropical Diseases

**DOI:** 10.3389/fchem.2021.719223

**Published:** 2021-06-16

**Authors:** Luciana Scotti, Eugene Muratov, Alejandro Speck-Planche, Marcus T. Scotti

**Affiliations:** ^1^ Postgraduate Program in Natural and Synthetic Bioactive Products, Federal University of Paraíba, João Pessoa, Brazil; ^2^ Laboratory for Molecular Modeling, The UNC Eshelman School of Pharmacy, University of North Carolina at Chapel Hill, Chapel Hill, NC, United States

**Keywords:** ligand-based virtual screening, structure-based virtual screening, machine learning, multi-target, artificial intelligence

Neglected Tropical Diseases (NTDs) are a group of infectious diseases that disproportionally affect impoverished countries. NTDs, because of their severity, negatively impact the disability-adjusted life year (DALY), a measure of overall disease burden on a country. This metric quantifies the potential years of life lost due to premature death as well as potential years of healthy life lost due to chronic states of illness and disability. NTDs, thus, transform the health of individuals and whole societies.

The world health organization estimates that NTDs affect more than one billion people in 149 countries and cost developing economies billions of dollars every year. In 2012, NTDs accounted for approximately 22 million DALYs globally. People suffering from these diseases often lose their abilities to contribute socially and economically, forgo educational opportunities, and are burdened with exorbitant expenses for treatment. NTDs are, therefore, a primary driver of the poverty cycle in many countries.

“Chemoinformatics has become an integral part of the drug discovery process, accelerating the search for novel chemicals with desired physicochemical, pharmacological, toxicological, and pharmacokinetic properties.”

This special issue of Frontiers in Chemistry is devoted to cheminformatics approaches in drug discovery for neglected tropical diseases. The papers that are included in our special issue cover a variety of research questions related to different aspects of computer-assisted drug discovery for NTDs. A brief description of these papers is provided below.

Choudhury and Bhardwaj report a dynamic hybrid pharmacophore model (DHPM), which represents the combined interaction features of different binding pockets. Mtb-DapB, a validated mycobacterial drug target, was used as a basis for model systems to explore the effectiveness of DHPMs to screen novel unexplored compounds. The model systems were subjected to 200 ns molecular dynamics simulations and trajectories were analyzed to identify stable ligand-receptor interaction features. Based on these interactions, conventional pharmacophore models (CPM) were generated from the individual binding sites while DHPMs were created from hybrid molecules occupying both binding sites. A huge library of 1,563,764 publicly available molecules was screened by CPMs and DHPMs. The screened hits obtained from both types of models were compared based on their Hashed binary molecular fingerprints and 4-point pharmacophore fingerprints using Tanimoto, Cosine, Dice, and Tversky similarity matrices. Molecules screened by DHPM exhibited significant structural diversity, better binding strength, and drug-like properties as compared to the compounds screened by CPMs indicating the efficiency of DHPM to explore new chemical space for anti-TB drug discovery. The idea of DHPM can be applied for a wide range of mycobacterial or other pathogen targets to venture into unexplored chemical space.

Vasconcelos and Rezende propose the use of integrated computational methods capable of evaluating the druggability of the predicted proteomes of *Leishmania braziliensis* and *Leishmania infantum*, species responsible for the different clinical manifestations of leishmaniasis in Brazil. The protein members of those proteomes were assessed based on their structural, chemical, and functional contexts applying methods that integrate data on molecular function, biological processes, subcellular localization, drug binding sites, druggability, and gene expression. These data were compared to those extracted from already known drug targets (BindingDB targets), which made it possible to evaluate *Leishmania* proteomes for their biological relevance and treatability. Through this methodology, the authors identified more than 100 proteins of each *Leishmania* species with druggability characteristics, and potential interaction with available drugs. Among those, 31 and 37 proteins of *L. braziliensis* and *L. infantum*, respectively, have never been tested as drug targets, and they have shown evidence of gene expression in the evolutionary stage of pharmacological interest. Also, some of those *Leishmania* targets showed an alignment similarity of <50% when compared to the human proteome, making these proteins pharmacologically attractive, as they present a reduced risk of side effects. The methodology used in this study also allowed the evaluation of opportunities for the repurposing of compounds as anti-leishmaniasis drugs, inferring potential interaction between *Leishmania* proteins and ∼1,000 compounds, of which only 15 have already been tested as a treatment for leishmaniasis.

Winkler reviewed the use of artificial intelligence and machine learning for the discovery of drugs to treat neglected tropical diseases. He discussed how computational methods are starting to make significant inroads into the discovery of drugs for neglected tropical diseases due to the increasing availability of large databases that can be used to train ML models, the ever-improving accuracy of these methods, the lower entry barrier for researchers, and widespread availability of public domain machine learning codes. He also summarized the application of artificial intelligence, largely the subset called machine learning, to modeling and prediction of biological activities and discovery of new drugs for neglected tropical diseases. The pathways for the development of machine learning methods in the short to medium term and the use of other artificial intelligence methods for drug discovery were discussed as well. The current roadblocks to, and likely impacts of, synergistic new technological developments on the use of ML methods for neglected tropical disease drug discovery in the future were also reviewed.

Kleandrova et al. report the first multi-target model based on quantitative structure-activity relationships and a multilayer perceptron neural network (mt-QSAR-MLP) to virtually design and predict versatile inhibitors of proteins involved in the survival and/or infectivity of different pathogenic parasites. The mt-QSAR-MLP model exhibited high accuracy (>80%) in both training and test sets for the classification/prediction of protein inhibitors. Several fragments were directly extracted from the physicochemical and structural interpretations of the molecular descriptors in the mt-QSAR-MLP model. Such interpretations enabled the generation of four molecules that were predicted as multi-target inhibitors against at least three of the five parasitic proteins reported here with two of the molecules being predicted to inhibit all the proteins. Docking calculations converged with the mt-QSAR-MLP model regarding the multi-target profile of the designed molecules. The designed molecules exhibited drug-like properties, complying with Lipinski’s rule of five, as well as Ghose’s filter and Veber’s guidelines.

